# Age and gender differences in the prevalence and patterns of multimorbidity in the older population

**DOI:** 10.1186/1471-2318-14-75

**Published:** 2014-06-17

**Authors:** José María Abad-Díez, Amaia Calderón-Larrañaga, Antonio Poncel-Falcó, Beatriz Poblador-Plou, José Manuel Calderón-Meza, Antoni Sicras-Mainar, Mercedes Clerencia-Sierra, Alexandra Prados-Torres

**Affiliations:** 1 Department of Health Wellbeing and Family, Government of Aragón, Zaragoza, Spain; 2 Department of Microbiology, Preventive Medicine and Public Health, University of Zaragoza, Zaragoza, Spain; 3 EpiChron Research Group on Chronic Diseases, Aragón Health Sciences Institute (IACS), IIS Aragón, Miguel Servet University Hospital, Via Universitas 36, 50017 Zaragoza, Spain; 4 Red de Investigación en Servicios de Salud en Enfermedades Crónicas (REDISSEC), Carlos III Health Institute, Zaragoza, Spain; 5 Teaching Unit of Preventive Medicine and Public Health, Aragón Health Sciences Institute (IACS), IIS Aragón, Zaragoza, Spain; 6 Zaragoza Sector III Primary Care Directorate, Aragon Health Service (SALUD), Zaragoza, Spain; 7 Planning Management, Badalona Serveis Assistencials S.A., Badalona, Spain; 8 Socio-Sanitary Assessment Unit, Miguel Servet University Hospital, Zaragoza, Spain

**Keywords:** Multimorbidity, Comorbidity, Chronic disease, Primary health care, Prevalence, Frail elderly

## Abstract

**Background:**

The coexistence of several chronic diseases in one same individual, known as multimorbidity, is an important challenge facing health care systems in developed countries. Recent studies have revealed the existence of multimorbidity patterns clustering systematically associated distinct clinical entities. We sought to describe age and gender differences in the prevalence and patterns of multimorbidity in men and women over 65 years.

**Methods:**

Observational retrospective multicentre study based on diagnostic information gathered from electronic medical records of 19 primary care centres in Aragon and Catalonia. Multimorbidity patterns were identified through exploratory factor analysis. We performed a descriptive analysis of previously obtained patterns (i.e. cardiometabolic (CM), mechanical (MEC) and psychogeriatric (PG)) and the diseases included in the patterns stratifying by sex and age group.

**Results:**

67.5% of the aged population suffered two or more chronic diseases. 32.2% of men and 45.3% of women were assigned to at least one specific pattern of multimorbidity, and 4.6% of men and 8% of women presented more than one pattern simultaneously. Among women over 65 years the most frequent pattern was the MEC pattern (33.3%), whereas among men it was the CM pattern (21.2%). While the prevalence of the CM and MEC patterns decreased with age, the PG pattern showed a higher prevalence in the older age groups.

**Conclusions:**

Significant gender differences were observed in the prevalence of multimorbidity patterns, women showing a higher prevalence of the MEC and PG patterns, as well as a higher degree of pattern overlapping, probably due to a higher life expectancy and/or worse health. Future studies on multimorbidity patterns should take into account these differences and, therefore, the study of multimorbidity and its impact should be stratified by age and sex.

## Background

The coexistence of two or more chronic health problems in the same person at one point in time, known as multimorbidity, is an important challenge facing health care systems in developed countries [1,2]. However, the dominant paradigm in medical research, training and care provision remains focused on a single disease approach, resulting in problems of coordination between primary and specialist care, and between routine and emergency care, for patients with multimorbidity [3]. Insufficient coordination of care derives in ineffective, inadequate and unsafe health care and generates dissatisfaction among patients and physicians [4]. Although multimorbidity is highly prevalent in all stages of life, it has major consequences for the older population [5]. In geriatric patients multimorbidity is linked to polypharmacy, frailty, health service misuse and lack of coordination [6], with consequences of increased mortality, frequency of adverse events, reduced quality of life and functional capacity, and stress on health care systems [7,8].

Several strategies have been proposed for delivering comprehensive care for older patients with multimorbidity, but the evidence on its effectiveness is limited [9]. The American Geriatrics Society recently set out guiding principles for the clinical management of this population, considering the multiple problems particular to each individual, their preferences and goals, the feasibility of the interventions, and the interactions among them [10,11].

Multimorbidity research has increased progressively [1], although the research community has not come to a consensus on how to measure it yet [12]. Thus, the prevalence of multimorbidity depends on its definition, the list of diseases considered, and even the source of information about diagnostics. Moreover, recent studies have revealed the existence of multimorbidity patterns clustering systematically associated health problems that fall beyond the standard concept of medical specialities established by health systems [13-19]. The clustering of diseases poses a challenge, both for the etiological research of chronic diseases and for the design of adequate prevention and treatment strategies. Still, the applicability of multimorbidity patterns in research and medical practice requires further knowledge of their prevalence, the diseases that are involved, their relationship with age, and the existence of potential gender differences.

In a previous study [13] we identified several clinically consistent patterns of multimorbidity in a primary care population, and found differences in the prevalence and clinical characteristics of these patterns by gender and age group. In this study we make a detailed analysis of such differences within the older population, describing the prevalence of these patterns of multimorbidity and of the diseases that are clustered in older men and women.

## Methods

### Design

This is an observational, retrospective, and multicentre study based on information gathered from the electronic health records (EHR) of primary care centres of two southern European regions of Spain: Aragon and Catalonia. The selection of centres participating in this study was based on the quality of the clinical information: (a) more than two years of experience in the use of EHR, (b) less than 20% of episodes with no diagnosis code, (c) less than 15% of entries with uncoded episodes, (d) less than 10% of prescriptions in uncoded episodes, (e) an average number of diagnoses per patient greater than 3.5, and (f) less than 10% of patients with no diagnostic information. From the initial 26 centres included in the dataset, we excluded 7 centres based on these criteria.

Patients included in the study were older than 64 years and seen at least once by their GP during 2008. The final study population was composed of 72,815 individuals from 19 urban health centres. For each of the included patient, the extracted data were age (later categorised in three groups: 65–74, 75–84 and ≥85), sex, and diagnostic episodes coded according to the International Classification of Primary Care (ICPC-2) [20].

This study was approved by the Ethics Committee of Clinical Research of Aragon (CEICA, for its initials in Spanish), who waived the need for written patient consent because the study was based on the statistical analysis of anonymous data.

### Multimorbidity patterns

To facilitate the handling of diagnostic information, health problems were grouped in Expanded Diagnosis Clusters (EDC) of the ACG® System [21]. To this end, ICPC codes were previously mapped into the International Classification of Diseases (ICD-9-CM) [22]. A chronic disease was defined as an illness lasting six months or more, including past illnesses requiring continuous care, diseases with risk of recurrence, or previous health problems that continued to affect the management of patients [23]. The Additional file 1 shows the included EDCs. To increase the epidemiological value of the study, only those EDCs with a prevalence above 1% were included.

Multimorbidity patterns were identified in a previous study [13] using exploratory factor analysis (see Additional file 1 for further methodological details). This technique enables identifying variables with a common underlying factor. The clinical plausibility of the identified patterns was assessed by three GPs and contrasted with the literature.

Three patterns of diseases were identified in the older population, and named as cardiometabolic (CM), psychogeriatric (PG) and mechanical (MEC). The diseases included in each of them are listed in Table 1.

**Table 1:** Diseases included in the multimorbidity patterns in the older population

**Pattern**	**Diseases**
**CM**	Atherosclerosis, Cardiac arrhythmia, Congestive heart failure, Diabetes, Gout, Hematologic disorders, Hypertension, Ischemic heart disease, Iron deficiency, Obesity, Other cardiovascular disorders
**MEC**	Anxiety and neuroses, Arthropathy, Cervical pain, Dermatitis y eczema, Disorders lipid metabolism, Diverticular disease colon, Gastroesophageal reflux, Low back pain, Osteoporosis, Prostatic hypertrophy, Thyroid disease, Varicose veins
**PG**	Behaviour problems, Cardiac arrhythmia, Cerebrovascular disease, Chronic ulcer of the skin, Congestive heart failure, Dementia and delirium, Iron deficiency, Osteoporosis, Parkinson’s disease

CM: cardiometabolic, MEC: mechanical, PG: psychogeriatric.

### Analysis

A descriptive analysis of the study variables was performed based on the calculation of frequencies and the graphical representation of the latter. The chi-square test was employed to test the association between sex and/or age and the prevalence of the patterns. Student’s t test was used to test the association between sex and/or age and the number of chronic diseases of a given multimorbidity pattern among individuals assigned to such pattern. STATA 12 was used for the statistical analysis and Excel 2007 for developing the graphs.

## Results

**Figure 1:**
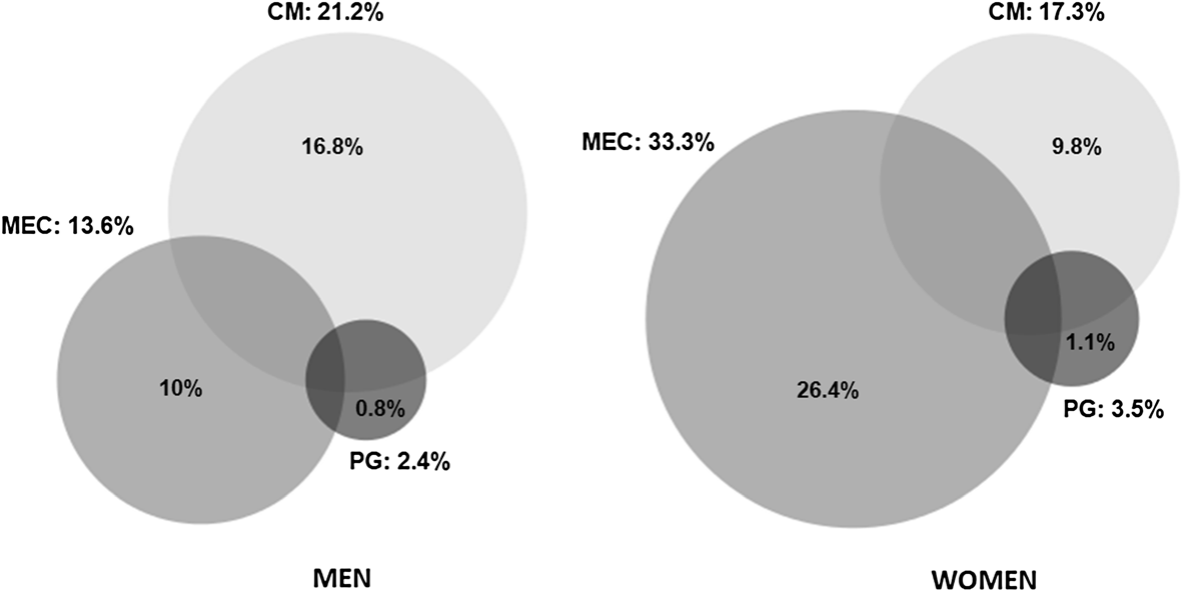
**Overlapping of multimorbidity patterns in men and women.** CM: cardiometabolic, MEC: mechanical, PG: psychogeriatric. Proportions inside the circles represent the frequency of non-overlapping disease patterns.

**Figure 2:**
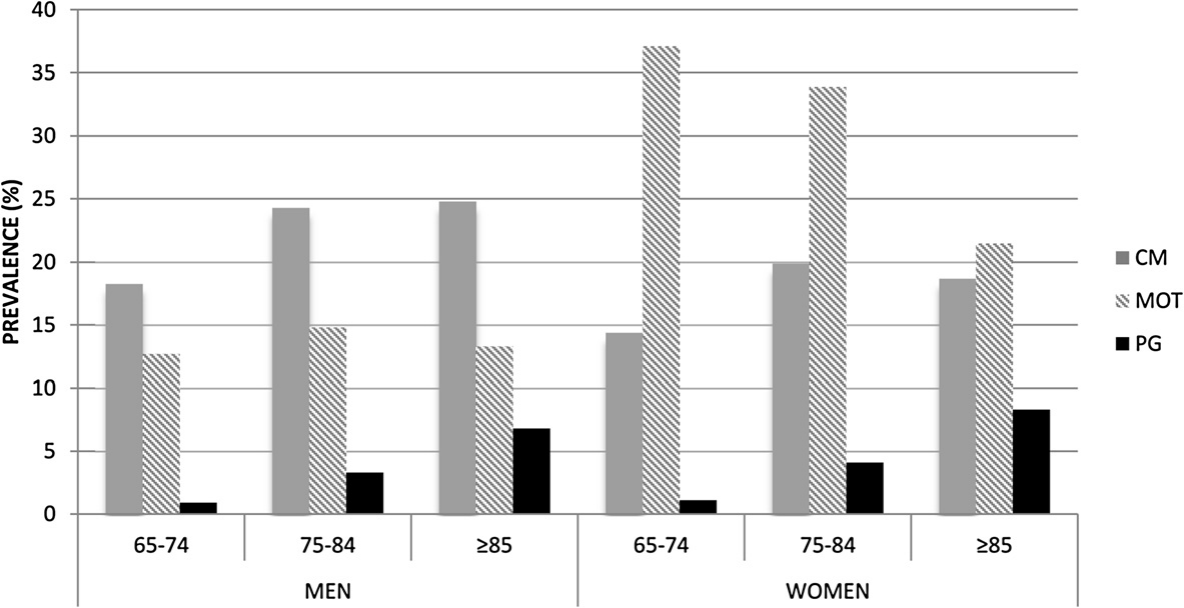
**Prevalence of multimorbidity patterns by sex and age groups.** CM: cardiometabolic, MEC: mechanical, PG: psychogeriatric.

A total of 72,815 patients over 64 years were included in the study; 13.6% of them were over 85 and 59.7% of them women. Overall, 67.5% of the patients had two or more chronic diseases simultaneously, this rate being even higher in women (69.3%) and in those aged 75–84 years (71.7%) (Table 2).In total, 32.2% of men and up to 45.3% of women were assigned to at least one pattern of multimorbidity (Figure 1). 27.6% of men and 37.3% of women were exclusively attributed to a unique pattern, and an overlapping between more than one pattern was seen in 4.6% of men and 8% of women.Whereas the CM pattern was 22.5% more prevalent in men than in women (21.2% vs. 17.3%, p < 0.001), the MEC pattern was more than double the prevalence in women compared to men (33.3% vs. 13.6%, p < 0.001). The PG pattern appeared simultaneously with the other two patterns in more than half of both men and women. The prevalence of the different patterns increased with age, except for the MEC pattern in women (Figure 2). There was also a decline in the prevalence of the CM pattern in women after the age of 85 years.

**Table 2:** Characteristics of the study population

	**Population**	**Mean number of diseases (SD)**	**Proportion with multimorbidity (CI 95%)**
**Total patients**	72,815 (100.0%)	2.5 (1.8)	67.5 (67.2-67.8)
**Age groups**			
**65**-**74**	34,283 (47.1%)	2.4 (1.7)	64.8 (64.3-65.3)
**75**-**84**	28,622 (39.3%)	2.7 (2.7)	71.7 (71.2-72.3)
≥**85**	9,910 (13.6%)	2.4 (1.9)	64.4 (63.5-65.3)
**Sex**			
**Men**	29,361 (40.3%)	2.4 (1.7)	64.8 (64.2-65.3)
**Women**	43,454 (59.7%)	2.6 (1.8)	69.3 (68.9-69.8)

Most individuals presented between two and three diseases of the corresponding pattern (Table 3). In general, men assigned to the CM pattern showed a significantly higher number of diseases of the corresponding pattern compared to women (p < 0.001). Conversely, women attributed to the MEC pattern presented a significantly higher number of coexisting diseases within the pattern (p < 0.001).

**Table 3:** Distribution of individuals assigned to each multimorbidity pattern according to the number of chronic diseases of the corresponding pattern by sex and age group

**Patterns**	**Number of diseases of the pattern**	**Men**				**Women**			
		**65-74**	**75-84**	**≥85**	**Total**	**65-74**	**75-84**	**≥85**	**Total**
		**(%)**	**(%)**	**(%)**	**(%)**	**(%)**	**(%)**	**(%)**	**(%)**
		**N** **=** **15,338**	**N** **=** **11,121**	**N** **=** **2,902**	**N** **=** **29,361**	**N** **=** **18,945**	**N** **=** **17,501**	**N** **=** **7,008**	**N** **=** **43,454**
**CM**	**2**	78.9	73.7	70.3	75.6	83.9	80.3	79.4	81.4
	**3**	17.2	20.9	24.3	19.6	13.8	16.8	17.9	15.9
	**≥4**	4	5.5	5.4	4.8	2.4	2.9	2.6	2.7
	**Mean** (**SD**)	2.3 (0.5)	2.3 (0.6)	2.4 (0.6)	2.3 (0.6)*	2.2 (0.5)	2.2 (0.5)	2.2 (0.5)	2.2 (0.5)*
**MEC**	**2**	78.5	76.7	77.7	77.7	58.8	60.8	66.4	60.4
	**3**	17.9	18.9	17.6	18.3	28.5	26.7	24	27.3
	**≥4**	3.6	4.4	4.7	4.2	12.7	12.5	9.6	12.4
	**Mean** (**SD**)	2.3 (0.5)	2.3 (0.6)	2.3 (0.6)	2.3 (0.5)*	2.6 (0.8)	2.6 (0.8)	2.5 (0.8)	2.6 (0.8)*
**PG**	**2**	89.6	86.2	80.2	85.1	90.1	86.5	81.8	85.2
	**3**	9.6	12.5	17.8	13.4	7.6	12.5	16.3	13.3
	**≥4**	0.7	1.4	2	1.4	2.4	0.9	1.9	1.6
	**Mean** (**SD**)	2.2 (0.4)	2.2 (0.4)	2.2 (0.5)	2.2 (0.4)	2.1 (0.4)	2.1 (0.4)	2.2 (0.4)	2.2 (0.4)

CM: cardiometabolic, MEC: mechanical, PG: psychogeriatric.

*Statistically significant differences between men and women (p < 0.001).

The most prevalent chronic diseases included in the CM pattern were hypertension, diabetes, and cardiac arrhythmia in both men and women, closely followed by obesity in women (Table 4). In the MEC pattern, the most prevalent diseases were prostatic hypertrophy, low back pain and arthropathy among men; and arthropathy, disorders of lipids, and lower back pain among women. In both sexes, a high prevalence was observed for several related health problems such as anxiety, neurosis, dermatitis and eczema. In patients with the PG pattern, the most prevalent diseases were iron deficiency, dementia and delirium, cerebrovascular disease, congestive heart failure and chronic skin ulcers. A high prevalence of cardiac arrhythmias was also observed in women.

**Table 4:** Prevalence of specific chronic diseases among individuals assigned to each multimorbidity pattern by sex and age group

**Patterns**	**Diseases included in the pattern**	**Men**				**Women**			
		**65-74**	**75-84**	≥**85**	**Total**	**65-74**	**75-84**	≥**85**	**Total**
		**(%)**	**(%)**	**(%)**	**(%)**	**(%)**	**(%)**	**(%)**	**(%)**
		**N** **=** **15,338**	**N** **=** **11,121**	**N** **=** **2,902**	**N** **=** **29,361**	**N** **=** **18,945**	**N** **=** **17,501**	**N** **=** **7,008**	**N** **=** **43,454**
**CM**	Atherosclerosis	10.3	12	11	11.1	---	---	---	---
	Cardiac arrhythmia	15.2	22.5	28.4	19.9	13.2	21.5	33.9	20.6
	Congestive heart failure	3	8.6	15.8	6.9	4.7	10	20.8	9.9
	Diabetes	62.6	56.4	45.2	57.9	63.1	59.1	49.2	58.8
	Gout	7.3	5.8	6.7	6.6	---	---	---	---
	Hematologic disorders	8.5	9.9	8.2	9.1	7.3	10.3	9.5	9.1
	Hypertension	86.5	82.9	77	83.8	92.2	91.6	88.6	91.3
	Ischemic heart disease	---	---	---	---	8.7	12.5	14.5	11.5
	Iron deficiency	9.9	17.7	29.3	15.5	---	---	---	---
	Obesity	15.7	7.9	5.4	11.1	29.8	18.1	7.4	20.4
	Other cardiovascular disorders	6.9	9.3	9.2	8.2	---	---	---	---
**MEC**	Anxiety and neuroses	25.6	24.3	32.9	25.8	31.9	33.7	38.8	33.4
	Arthropathy	45.8	49	42.8	46.9	40.8	47	46.5	43.9
	Cervical pain	12.2	12.4	7.3	11.8	9.9	8.1	5.7	8.7
	Dermatitis y eczema	31.2	28.7	32.9	30.3	15	15.3	18.1	15.4
	Disorders lipid metabolism	---	---	---	---	45.8	38.8	32.2	41.5
	Diverticular disease colon	---	---	---	---	2.6	3.2	4.5	3
	Gastroesophageal reflux	10.2	10.1	9.6	10.1	7.5	8.6	9.1	8.1
	Low back pain	48.5	47.1	40.4	47.2	38.7	38.2	35.4	38.2
	Osteoporosis	4.6	6.3	8.3	5.7	32.5	28.9	22.3	30
	Prostatic hypertrophy	47.4	50.3	53.4	49.2	---	---	---	---
	Thyroid disease	---	---	---	---	17.9	15.2	14.4	16.4
	Varicose veins	---	---	---	---	15.5	18.6	19.5	17.2
**PG**	Behaviour problems	16.3	12.5	15.7	14.1	---	---	---	---
	Cardiac arrhythmia	---	---	---	---	50.7	45.7	42	45
	Cerebrovascular disease	46.7	36.6	32	37.2	25.1	26.5	25.4	25.9
	Chronic ulcer of the skin	17	23.6	21.8	21.8	17.5	17.3	29.9	22.2
	Congestive heart failure	17.8	28.3	30	26.7	30.3	30.1	27.1	29
	Dementia and delirium	31.1	39.1	48.2	40.1	38.4	44.4	49.9	45.7
	Iron deficiency	48.9	49.3	47.7	48.8	50.2	50.7	45.8	48.7
	Osteoporosis	17.8	11.4	9.6	12.1	---	---	---	---
	Parkinson’s disease	15.6	14.7	16.8	15.4	---	---	---	---

CM: cardiometabolic, MEC: mechanical, PG: psychogeriatric.

There were differences in the diseases included in the patterns among men and women (Table 4). In the CM pattern, atherosclerosis, gout and/or iron deficiency were associated with the pattern in men but not in women, whereas ischemic heart disease was associated with the pattern only in women. In the MEC pattern, diverticular disease of the colon, thyroid disease, varicose veins and, remarkably, disorders of the lipid metabolism were associated with the rest of the disease of the pattern only in women. In the PG pattern, osteoporosis, Parkinson’s disease and behaviour problems were clustered in the pattern only in men, whereas cardiac arrhythmia was associated only in women.

## Discussion

Almost seven out of 10 individuals aged 65 years and older had two or more chronic diseases. Around 80% of them were assigned to at least one multimorbidity pattern and 12% to at least two. The study of disease patterns is gaining increasing interest, under the assumption that some diseases are systematically associated with each other beyond randomness [12]. The current definition of multimorbidity is simple but too broad and unspecific, hindering a better understanding of the phenomenon [23] and the translation of the emergent knowledge on the nature of multimorbidity and its negative effects into clinical decision and management tools [24]. Moreover, probably many associations among two or more diseases have yet to be uncovered, as they may be prognostic of specific health outcomes [25].

In this study we gained insight into the epidemiology of previously identified multimorbidity patterns in the older male and female population. While the prevalence of the PG pattern increased with age, the frequency of the other two patterns identified in this population group decreased with age, especially among those over 85. We hypothesize that this fact, previously described [26], can be related to the increased survival of people without multimorbidity, less thorough diagnostic efforts in the major older population, and/or recording bias in this age group.

There were gender differences in the prevalence of multimorbidity and of specific disease patterns, women showing a higher prevalence of the MEC and PG patterns as well as a higher degree of pattern overlapping. Other studies have described a greater prevalence of multimorbidity among females, relating this finding to a longer life expectancy and worse health status compared to males [14,17,19]. However, the prevalence of the CM pattern was lower in women. The existence of gender disparities in the diagnosis and treatment of cardiovascular diseases has been discussed previously [27,28]. According to these studies, there are gender differences in the early detection, referral and treatment of cardiovascular diseases, leading to less intensive diagnostic procedures, a higher probability of delayed treatment, and increased risk of emergency admissions and worse outcomes among the female population. Results of this study suggest that these disparities may be even higher in over-aged populations since GPs may be less prone to diagnose and treat cardiovascular diseases in these very aged female patients.

The CM pattern has been repeatedly identified in previous studies and through a recent systematic review [14-19]. Unexpectedly, in our study, dyslipidemia was included as part of this pattern in persons under 65 years but not in the more aged groups. The prevalence of this risk factor in our population decreased with age, which is consistent with other studies in our context. For example, in the 2006 National Health Survey, the prevalence of hypercholesterolaemia went from 30.9% in the age group of 65–74 years down to 22.3% in those over 75 years [29]. This decrease in the diagnosed prevalence of dyslipidemia might explain the dropping out of the latter from the CM pattern among older patients. The controversy around the role of dyslipidemia in cardiovascular mortality or the appropriate target levels of cholesterol in clinical guidelines could explain such decrease [30].

The associations between the diseases included in the MEC pattern (i.e. joint pain, anxiety, and neurosis and somatoform disorders) have been described previously [14,17,19]. Its prevalence was higher in women and varied in an inverted U shape fashion with age (i.e. decreased prevalence from 85 years onwards). Our study did not include institutionalized people which could lead to an underrepresentation of patients with advanced mechanical problems. Older women that presented this pattern had a higher mean number of diseases than men. This could be related to a greater number of diseases included in the pattern among women or to a worse health. It is to be remarked that some diseases with a high prevalence in women are not included in the pattern in men.

The PG pattern showed disease interactions already described in the literature [14,31,32], as is the case of the co-occurrence of dementia and other age-related diseases (i.e. cerebrovascular disease and heart failure). The frequency of this pattern significantly increased from 85 years onwards, and it showed an important overlapping with the other patterns. The PG pattern, characteristic of the very aged, seems to cluster several frailty-related problems. Although the lack of a standard definition of frailty [33], it has been related to an increased vulnerability to adverse outcomes, and has been modelled as an accumulation of deficits.

Regarding the gender differences related to the diseases conforming each of the three patterns, these could be partly explained by the differences in the prevalence of diseases among men and women. This was the case of gout and Parkinson’s disease which showed prevalence lower than 1% in women, and were therefore not included in the factor analysis in older females. Another reason for such gender disparities was related with the factor score threshold established when identifying the patterns (i.e. 0.25), which resulted in the exclusion of certain diseases with values slightly below this cut-off. Such was the case of the ischemic heart disease among male patients with the CM pattern, thyroid disease among men with the MEC pattern, and/or the behaviour problems among women with the PG pattern. Still, a couple of findings deserve further confirmation in future studies. Such was the case of the clustering, only in men, of osteoporosis within the PG pattern and/or of atherosclerosis within the CM pattern. The higher prevalence of cerebrovascular diseases and its associated treatment with antiplatelet agents and proton-pump inhibitors could explain the incorporation of osteoporosis in men [34]. Regarding the absence of atherosclerosis in women, it could be explained by their lower smoking rates.

### Strengths and limitations

This study was based on diagnostic information gathered from medical records collected during patients’ visits to primary care. In our context, the registry of diagnoses in medical records is not linked to billing systems or financial incentives, and there is no limit regarding the number of diagnoses listed per patient. This provides rigour to our findings, as it avoids selection bias and leads to more representative results compared to those obtained from survey-based studies [15].

Conversely, the completeness of the information could be limited due to the workload of GPs and the characteristics of the coding system (ICPC). Therefore, the frequency of certain health problems might be underestimated. We suspect that this could be the case of some lifestyle risk factors, such as smoking. However, this bias is minimised by the fact that this study focused on chronic diseases with stable and long-term diagnostic coding. This limitation was further controlled by the data quality criteria established in the selection of health centres. Another problem regarding the diagnostic accuracy may arise from a potential underestimation of the prevalence of diseases routinely diagnosed and treated in the specialized care (i.e. cancer or rare diseases).

The exclusion of health centres based on documentation quality may have introduced bias if this was related to population factors such as health status or deprivation. Although we cannot rule out the possibility of this bias, we think it is unlikely to have occurred in our data as the distribution of these characteristics is similar among the included centres and the total population. The study does not cover rural areas and it is possible that results may be different in the rural environment [35].

It is worth noting that this study was based on the concept of chronic disease, and the boundary between “acute” and “chronic” disorders is not always clear, as pointed out by Starfield [36]. Moreover, there may be other type of problems affecting patients’ quality of life but which may not be considered by the physician.

## Conclusions

The results of this study show the existence of significant gender differences in the presentation of multimorbidity patterns between men and women, women showing a higher prevalence of the MEC and PG patterns but a lower prevalence of the CM pattern. Moreover, a higher degree of pattern overlapping was observed among women, probably due to a higher life expectancy and/or worse health. Regarding age, while the prevalence of the CM and MEC patterns decreased with age, the PG pattern was most prevalent in the oldest age group.

These differences are not always adequately reported in the literature and require further research, especially in the female population. Future studies on multimorbidity patterns should take into account these differences and, therefore, the study of multimorbidity and its impact should be stratified by age and sex.

## Competing interests

The authors declare that they have no competing interests.

## Authors’ contributions

JMAD, ACL and APT took part in the design and conduct of the study. JMAD was responsible for drafting the manuscript. BPP, APF and ASM performed the data extraction and prepared databases. JMAD, ACL, BPP, APT and JMCM performed the analysis and interpretation of the data. All the authors reviewed and provided input for the final version of the manuscript.

## Pre-publication history

The pre-publication history for this paper can be accessed here:

http://www.biomedcentral.com/1471-2318/14/75/prepub

## Supplementary Material

Additional file 1 **Prevalence of Expanded Diagnostic Clusters by sex and age group.** Methodological details of the exploratory factor analysis. Click here for file
