# Supplementary material for: Age and gender differences in the prevalence and patterns of multimorbidity in the older population
Source: BMC Geriatr. 2014 Jun 17;14:75. doi: 10.1186/1471-2318-14-75 (PMC4070347; doi:10.1186/1471-2318-14-75)
Supplement: Additional file 1 — Prevalence of Expanded Diagnostic Clusters by sex and age group. Methodological details of the exploratory factor analysis. [file 1471-2318-14-75-S1.doc]

**APPENDIX I Prevalence of Expanded Diagnostic Clusters by sex and age group**

| **EDC** | **Description** | **Men** | | | | **Women** | | | | **Total** |
| --- | --- | --- | --- | --- | --- | --- | --- | --- | --- | --- |
|  |  | **65-74** | **75-84** | **>85** | **Total** | **65-74** | **75-84** | **>85** | **Total** |  |
|  |  | **(%)** | **(%)** | **(%)** | **(%)** | **(%)** | **(%)** | **(%)** | **(%)** | **(%)** |
| ALL04 | Asthma | 1.4 | 1.1 | 1.2 | 1.3 | 3.1 | 2.9 | 2.1 | 2.9 | 2.2 |
| ALL06 | Disorders immune system | 0.2 | 0.3 | 0.2 | 0.3 | 0.3 | 0.4 | 0.2 | 0.3 | 0.3 |
| CAR03 | Ischemic heart disease | 4.6 | 6.6 | 6.2 | 5.5 | 1.8 | 3.4 | 3.9 | 2.8 | 3.9 |
| CAR05 | Congestive heart failure | 0.7 | 2.5 | 5.1 | 0.5 | 0.8 | 2.7 | 5.6 | 0.7 | 0.6 |
| CAR06 | Cardiac valve disorders | 0.7 | 0.9 | 0.7 | 0.8 | 0.7 | 1.2 | 0.9 | 1.0 | 0.9 |
| CAR09 | Cardiac arrhythmia | 4.0 | 7.4 | 10.1 | 5.9 | 2.9 | 5.8 | 9.0 | 5.0 | 5.4 |
| CAR10 | Atherosclerosis | 2.8 | 3.8 | 3.9 | 3.3 | 0.8 | 1.5 | 1.8 | 1.2 | 2.1 |
| CAR11 | Disorders lipid metabolism | 21.2 | 15.2 | 9.0 | 17.7 | 24.5 | 19.6 | 10.6 | 20.3 | 19.3 |
| CAR12 | Acute myocardial infarction | 2.9 | 3.7 | 3.1 | 3.2 | 0.5 | 1.2 | 1.3 | 0.9 | 1.9 |
| CAR14 | Hypertension | 39.0 | 44.0 | 40.0 | 41.0 | 40.0 | 51.8 | 47.0 | 45.9 | 43.9 |
| CAR16 | Other cardiovascular disorders | 2.0 | 3.2 | 3.0 | 2.6 | 2.5 | 3.3 | 3.2 | 2.9 | 2.8 |
| EAR08 | Deafness, hearing loss | 1.7 | 1.8 | 2.1 | 1.8 | 1.5 | 2.1 | 1.7 | 1.8 | 1.8 |
| END02 | Osteoporosis | 0.9 | 1.4 | 1.5 | 1.2 | 16.0 | 12.8 | 7.1 | 13.3 | 8.4 |
| END04 | Thyroid disease | 2.6 | 3.1 | 3.2 | 2.8 | 8.7 | 6.7 | 4.6 | 7.2 | 5.4 |
| END05 | Other endocrine disorders | 0.7 | 0.7 | 0.8 | 0.8 | 1.7 | 1.5 | 1.0 | 1.5 | 1.2 |
| END06 | Diabetes | 19.4 | 20.8 | 17.2 | 19.7 | 13.7 | 16.6 | 13.2 | 14.8 | 16.8 |
| EYE03 | Retinal disorders | 0.9 | 0.7 | 1.1 | 0.8 | 0.7 | 1.0 | 0.8 | 0.9 | 0.8 |
| EYE02 06 | Blindness, cataract, aphakia | 4.7 | 8.3 | 7.4 | 6.3 | 5.7 | 9.9 | 7.0 | 7.6 | 7.1 |
| EYE08 | Glaucoma | 3.1 | 4.0 | 3.7 | 3.5 | 3.5 | 4.7 | 3.9 | 4.1 | 3.8 |
| FRE12 | Utero-vaginal prolapse | --- | --- | --- | --- | 1.1 | 0.8 | 0.5 | 0.9 | 0.9 |
| GAS05 | Chronic liver disease | 1.3 | 0.6 | 0.7 | 1.0 | 1.0 | 0.8 | 0.3 | 0.8 | 0.9 |
| GAS08 | Gastroesophageal reflux | 2.4 | 2.5 | 2.3 | 2.4 | 3.4 | 3.6 | 2.8 | 3.4 | 3.0 |
| GAS02 09 10 | Chronic intestinal disorders | 1.1 | 1.4 | 1.4 | 1.3 | 2.0 | 2.2 | 1.8 | 2.0 | 1.7 |
| GSU08 | Varicose veins lower extremities | 1.9 | 2.6 | 2.5 | 2.2 | 7.4 | 8.2 | 6.6 | 7.6 | 5.4 |
| GUR04 | Prostatic hypertrophy | 14.3 | 16.4 | 14.6 | 15.1 | --- | --- | --- | --- | 15.1 |
| GUR09 | Renal calculi | 1.3 | 0.8 | 0.3 | 1.0 | 0.8 | 0.6 | 0.2 | 0.6 | 0.8 |
| GUR10 | Prostatitis | 0.8 | 0.6 | 0.4 | 1.0 | --- | --- | --- | --- | 1.0 |
| HEM02 | Iron deficiency | 2.5 | 5.7 | 9.9 | 4.5 | 3.5 | 7.1 | 10.3 | 6.1 | 5.4 |
| HEM03 | Thrombophlebitis | 0.7 | 0.9 | 1.1 | 0.8 | 0.8 | 1.2 | 1.0 | 1.0 | 0.9 |
| HEM08 | Hematologic disorders | 2.3 | 3.1 | 2.7 | 2.6 | 1.7 | 2.6 | 2.3 | 2.1 | 2.3 |
| MAL01-05 08 10-15 18 | Malignancies | 6.6 | 9.4 | 10.0 | 8.0 | 3.8 | 3.9 | 3.8 | 3.8 | 5.5 |
| MUS03 | Arthropathy | 12.9 | 14.9 | 13.1 | 13.7 | 21.3 | 23.5 | 17.4 | 21.6 | 18.4 |
| MUS13 | Cervical pain | 2.8 | 3.0 | 2.0 | 2.8 | 4.6 | 3.7 | 2.0 | 3.8 | 3.4 |
| MUS14 | Low back pain | 13.8 | 13.7 | 10.9 | 13.4 | 19.7 | 18.4 | 12.5 | 18.0 | 16.2 |
| NUR03 | Peripheral neuropathy | 1.3 | 1.4 | 1.5 | 1.4 | 1.9 | 1.9 | 1.4 | 1.8 | 1.7 |
| NUR05 | Cerebrovascular disease | 2.6 | 4.7 | 6.6 | 3.8 | 0.3 | 3.1 | 5.3 | 2.7 | 3.1 |
| NUR06 | Parkinson’s disease | 0.8 | 1.8 | 1.8 | 1.3 | 0.6 | 1.3 | 1.4 | 1.0 | 1.1 |
| NUR11 | Dementia and delirium | 2.0 | 5.4 | 9.6 | 4.0 | 3.0 | 7.8 | 12.2 | 6.4 | 5.5 |
| NUR 07 08 16 17 19 21 | Neurologic disorders | 1.2 | 1.4 | 0.9 | 1.2 | 1.1 | 1.1 | 0.7 | 1.0 | 1.1 |
| NUT03 | Obesity | 3.8 | 2.5 | 1.8 | 3.1 | 6.6 | 4.6 | 1.7 | 5.0 | 4.2 |
| PSY01 | Anxiety and neuroses | 6.3 | 6.8 | 7.8 | 6.6 | 15.6 | 15.7 | 12.8 | 15.2 | 11.7 |
| PSY02 | Substance use | 1.2 | 0.5 | 0.2 | 0.8 | 0.2 | 0.1 | 0.0 | 0.1 | 0.4 |
| PSY04 | Behaviour problems | 0.9 | 1.3 | 2.2 | 1.2 | 1.8 | 2.2 | 2.5 | 2.0 | 1.7 |
| PSY05 07 08 | Other mental health disorders | 0.6 | 0.5 | 0.6 | 0.6 | 0.9 | 0.6 | 0.3 | 0.7 | 0.6 |
| PSY09 | Depression | 0.7 | 0.8 | 0.6 | 0.7 | 2.1 | 2.1 | 1.2 | 2.0 | 1.5 |
| REC03 | Chronic ulcer of the skin | 0.5 | 1.5 | 3.3 | 1.1 | 0.5 | 1.6 | 4.8 | 1.7 | 1.5 |
| RES04 | Emphysema, chronic bronchitis, COPD | 7.9 | 11.3 | 11.9 | 9.6 | 1.9 | 2.8 | 2.7 | 2.4 | 5.3 |
| RHU02 | Gout | 2.1 | 1.9 | 2.0 | 2.0 | 0.3 | 0.4 | 0.4 | 0.3 | 1.0 |
| SKN02 | Dermatitis, eczema | 7.5 | 7.9 | 8.6 | 7.8 | 7.0 | 6.7 | 5.7 | 6.7 | 7.1 |
| SKN12 | Psoriasis | 1.2 | 1.1 | 0.2 | 1.1 | 0.8 | 0.6 | 0.3 | 0.6 | 0.8 |
| SKN13 | Disease of hair and hair follicles | 0.2 | 0.2 | 0.3 | 0.2 | 1.2 | 0.7 | 0.4 | 0.9 | 0.6 |

**APPENDIX II Methodological details of the exploratory factor analysis**

**Statistical analysis of the patterns**

The clustering of diseases into multimorbidity patterns was studied by means of an exploratory factor analysis. This technique was chosen because, in addition to identifying associations among groups of variables, it allows for variables to be included in more than one pattern simultaneously.

The factor analysis was based on a correlation matrix to determine which diagnostic variables comprised each factor. To this end, EDCs were coded in a binary format (i.e., 0=no disease and 1=presence of the disease). Due to the dichotomous nature of the variables, tetra-choric correlation matrices were used[[1]](#footnote-2). In doing so, it was assumed that dichotomous diagnoses had underlying, continuous characteristics. That is, we assumed that the chronic diseases included in our analysis had a progressive course (i.e., the accumulation of risk factors before the onset and/or the progression after the onset of the disease) and were diagnosed during this course if they reached a certain threshold.

The factors resulting from this analysis were interpreted as multimorbidity patterns (i.e., EDCs frequently related to each other), and each factor score, which had a value between -1 and 1, represented the association of each of the diagnoses with its disease pattern. For determining which EDCs formed each multimorbidity pattern, those with scores equal to or greater than 0.25 for each factor were selected.

The extraction of the disease factors was performed using the principal factor method, and it was assumed that the extracted factors did not explain the total variance of the analysed EDCs. To determine the number of factors to extract, a scree plot was utilised in which the eigenvalues of the correlation matrix were represented in descending order. The number of factors extracted corresponds to the sequence number of the eigenvalue that produces the inflection point of the curve[[2]](#footnote-3). In some situations, the Heywood phenomenon was observed due to the uniquely negative scores, which produced factor scores greater than 1. This can occur when a small or excessive number of factors are removed. In this case, as well as when a clear solution was not obtained by the scree plot, a clinical evaluation of different solutions was conducted. To facilitate the interpretation of the factors, an oblique rotation (Oblimin) was applied.

The adequacy of the sample used to perform the factor analysis was analysed by measuring the Kaiser-Meyer-Olkin (KMO) for each age and sex group. This parameter takes values between 0 and 1, which are closer to 1 with a greater goodness of fit. In addition, as a measure of model goodness-of-fit, the proportion of cumulative variance was obtained, which describes the variability of the diagnostic data explained by the patterns.

Given that multimorbidity is defined as the simultaneous presence of two or more chronic diseases, it was agreed that an individual had a specific pattern of multimorbidity if he/she presented with at least two of the diseases that comprised the pattern. Ultimately, this permitted the calculation of the prevalence of each of the multimorbidity patterns.

**Clinical consistency of the patterns**

Clinical relevance was studied in three phases. First, two qualified primary care physicians with research and clinical experience independently reviewed the clinical plausibility of disease interactions. Next, a joint analysis of both independent reports was performed by a third physician specialized in public health and experienced in health services research, identifying agreements and disagreements between both family physicians. Last, results were resubmitted to the primary care professionals and discussed in a final consensus meeting. The main arguments and conclusions were also contrasted with a literature review by other members of the research team.

1. Kubinger KD (2003) On artificial results due to using factor analysis for dichotomous variables. Psycology Science 45: 106–110. [↑](#footnote-ref-2)
2. Tabchnic B, Fidell L (2006) Using Multivariate Statistics. Boston: Allyin & Bacon. [↑](#footnote-ref-3)
